# Gendered difference in motivational profiles, achievement, and STEM aspiration of elementary school students

**DOI:** 10.3389/fpsyg.2022.954325

**Published:** 2022-08-29

**Authors:** Kezia Olive, Xin Tang, Anni Loukomies, Kalle Juuti, Katariina Salmela-Aro

**Affiliations:** Faculty of Educational Sciences, University of Helsinki, Helsinki, Finland

**Keywords:** motivation, elementary school, expectancy-value theory, gender, STEM aspiration, latent profile analysis, latent transition analysis

## Abstract

To better understand the gender gap in science, technology, engineering and math (STEM) aspiration, the article examines the critical role of domain-specific motivation (i.e., expectancy and task values). Using longitudinal data from 5th and 6th grade (∼11–12-year-old) students (*n* = 360, 55% girls), person-oriented analyses was applied to understand the gendered motivational profiles and their longitudinal influence on achievement and STEM aspiration. Specifically, we aimed to (1) derive motivational belief profiles regarding science, mathematics, and language (Finnish), (2) analyze the stability and change in the profiles between the 5th and 6th grade, (3) assess the relationship between motivational profiles and achievement and STEM aspiration, and (4) test for gender differences. We derived four motivational profiles for both years: high motivation in all subjects (∼21%), high mathematics motivation (∼46%), low mathematics motivation (∼11%), and low motivation in all subjects (∼8%). Latent transition analysis revealed that most students remained in the same profile throughout the 2 years. We found evidence of gendered differences in the motivational profiles and the chance of transitioning between profiles. More girls are characterized by low math motivation, while boys are more likely to transition to higher math motivation in 6th grade. The motivational difference is reflected in their achievement, although not strongly coupled with their STEM aspiration. The findings suggest that at this developmental stage, Finnish students have not developed a strong association between (gendered) STEM aspiration and their domain-specific motivation, although their motivation may have influenced their achievement. Interpretation and practical implications are discussed.

## Introduction

As part of the continuous effort to narrow the gender gap in science, technology, engineering, and mathematics (STEM), it is crucial to understand the factors that link gender and STEM involvement. Wang and Degol (2017) have discussed the various factors linking gender to differing levels of STEM engagement, and students’ motivational beliefs are one of the most significant factors. Most scholars studying motivational beliefs and their relationship to gender and STEM involvement have been guided by situated expectancy value theory (SEVT) (Eccles et al., 1983; Eccles and Wigfield, 2020) and dimensional comparison theory (DCT) (Moller and Marsh, 2013). The SEVT framework provides a foundation for how personal characteristics, such as gender, influence students’ motivational beliefs and academic outcomes. The DCT framework explains this process further, as it posits that each student will make internal comparisons between domains, which also significantly shapes their motivational beliefs (Moller and Marsh, 2013; Wigfield et al., 2020). Taken together, it is important to investigate motivational beliefs and their critical role in gendered STEM participation.

Yet, the relationship between gender, motivational beliefs, and STEM participation is less understood in elementary school students, even though the SEVT and DCT models indicate that motivational beliefs change over time (Eccles et al., 1993; Eccles and Wigfield, 1995; Guo et al., 2018b; Wan et al., 2021; Tang et al., 2022). Studies have found that students’ beliefs about themselves and about different fields, such as STEM, are found to develop incrementally (Watson and McMahon, 2005; van Tuijl and van der Molen, 2015). During years prior to high school, students’ interest and early educational experiences, especially in math and science, already sets the stage for their exploration and perceptions which predicts the subsequent choices they make (Hartung et al., 2005; Pinxten et al., 2012; Wang and Degol, 2017). These findings highlight the necessity of understanding the early years of students’ motivational development and linking it to the factors influencing their outcomes.

Thus far, however, most studies that have addressed students’ motivational beliefs and STEM aspiration have focused on data collected from adolescents (Gaspard et al., 2018, 2019; Guo et al., 2018b; Hsieh et al., 2019; Lazarides et al., 2021). Of the limited number of studies addressing elementary school students, researchers have examined only a few sets of domains and motivational constructs (Nurmi and Aunola, 2005; Gottfried et al., 2013; Musu-Gillette et al., 2015; Viljaranta et al., 2016; Petersen and Hyde, 2017). Consequently, there is a gap in our understanding of (gendered) motivational development given the limited number of studies involving elementary school students.

In this study, we aim to further understand the relationship between gender and STEM-related achievement and aspiration by examining the motivational beliefs of elementary school students in various subject domains. More specifically, we focus on students at the end of elementary school, right before they transition to (lower) secondary school, or junior high school. This will help to capture the transition period associated with significant changes in motivational beliefs (Watt, 2004) and offer new insights on the meaningful time frames in students’ motivational belief development and its association with gender and important educational outcomes.

### Theories on motivational beliefs

Situated expectancy value theory (SEVT) (Eccles et al., 1983; Eccles and Wigfield, 2020) focuses on an individual’s motivational beliefs, processes of gender socialization, and choice behaviors. With this theory, motivational beliefs are conceptualized as a student’s subjective task values (consisting of intrinsic value, utility value, attainment value, and cost) and expectations of success. *Intrinsic value* refers to the internal drive or enjoyment that a person has for a certain topic; *utility value* describes the future instrumental possibility of a certain behavior resulting in a particular goal (e.g., being good at math will help them in applying for an engineering degree); *attainment value* focuses on how a person attributes the importance of a certain behavior to their perceived identity (e.g., it is important for a girl like me to have good grades in languages); and *cost* refers to the perceived negative consequences for a person engaging with a certain task or behavior. In addition to addressing these values, the framework also described a student’s expectations through *self-concept of ability*, which is an individual’s perception of or belief about their ability level in a certain subject or domain (i.e., am I good at this? Can I really do this task?). The framework also described the influence of gender on an individual’s hierarchy of values—the likelihood that an individual’s personal characteristics, such as gender, will influence the hierarchy of their values, expectations, and choice behaviors (Eccles et al., 1993; Eccles, 2009).

Dimensional comparison theory (DCT) explains how these inner hierarchies develop across different domains or subjects in school (Moller and Marsh, 2013; Jansen et al., 2015). This theory proposes that students will compare their perceived performance in similar (e.g., math and science) and dissimilar domains (e.g., languages and math). They will then use the comparison to shape their motivational beliefs, including subjective task value hierarchies and expectancies in specific domains (Wigfield et al., 2020).

The combination of these theories has shown that there is a unique and rich process of motivational beliefs development in different domains within each individual. Both theories highlight the need to track the intra-individual hierarchy difference among students over time, a finding confirmed by different person-oriented studies (see a review by Wigfield and Eccles, 2020).

### Motivational beliefs development

Another important assumption of the SEVT and DCT models is that students’ motivational beliefs develop over time, though most studies to date have focused only on the motivational beliefs of adolescents (e.g., Guo et al., 2018b; Gaspard et al., 2019; Lazarides et al., 2021). The focus on an adolescent timeframe is understandable, as students become more and more stable in their differentiation of interests, confidence, and achievement in specific domains during those years (e.g., Gaspard et al., 2018, 2019; Lazarides et al., 2021; Wan et al., 2021). However, understanding the development of elementary school students is no less critical.

Elementary school students’ motivational beliefs are an important foundation for further development, even if they are not as well differentiated as adolescents. As Eccles et al. (1993; Wigfield, 1994) have suggested, until the 5th grade, students have only developed a full understanding of the intrinsic value construct. They discovered that a full understanding of the other types of task values only start after this point. Nevertheless, studies conducted with students below 5th grade (which examined only their intrinsic values) still found domain-specific differences (Nurmi and Aunola, 2005; Viljaranta et al., 2016; Oppermann et al., 2021). Other studies with a longer time span and more task value dimensions have also confirmed persistent differences in intra-individual hierarchies of motivational beliefs starting from elementary school age (Archambault et al., 2010; Musu-Gillette et al., 2015). Furthermore, results from previous studies have also suggested that task value differences may begin in these early years and become the foundation for greater motivational gaps in older students (Guo et al., 2018b; Muenks et al., 2018).

Despite the suggestion that elementary school students’ motivational beliefs are important foundation for further development of motivational belief, empirical evidence that clearly demonstrate this process is still scarce. Most longitudinal studies have only examined motivational beliefs in few domains independently, such as literacy (e.g., Archambault et al., 2010), mathematics (e.g., Musu-Gillette et al., 2015), or science (e.g., Vinni-Laakso et al., 2019). Even though several studies have considered more domains, they focused only on limited motivational constructs. For example, they mainly focused on the intrinsic motivation of students in the first years of schooling (Nurmi and Aunola, 2005; Oppermann et al., 2021). This highlights the need to provide more insight into the development of elementary school students’ motivational beliefs in various domains and with respect to a comprehensive list of motivational belief constructs.

### Motivational beliefs and academic outcomes

Different studies have confirmed that students’ motivational profiles in different subjects indeed predict their academic outcomes, including achievement and STEM aspiration (for a review, see Wigfield et al., 2009). Scholars have found that differentiation of domain-specific motivation intensify during secondary school (Chow et al., 2012; Guo et al., 2018b; Lazarides et al., 2021; Wan et al., 2021) and predict students’ subsequent achievement in the said domain (Bong et al., 2012; Safavian and Conley, 2016). The domain-specific mapping of motivation and achievement has also been observed in younger students (Nurmi and Aunola, 2005; Viljaranta et al., 2016).

Domain-specific motivational beliefs influence not only achievement but also students’ future STEM career aspirations. Adolescent students who are characterized by a higher level of motivation in mathematics were more likely to aspire to a career in physical science and information technology (Chow et al., 2012; Guo et al., 2018b). Such students are also more likely to end up choosing a STEM-related career (Wang and Degol, 2013; Wang et al., 2015; Guo et al., 2018a). Studies with elementary school students have also yielded similar results, with higher science and math-related values predicting future STEM aspiration (Vinni-Laakso et al., 2019; Oppermann et al., 2021). This finding is in line with general findings showing that students’ adult career choices (especially in STEM) are significantly influenced by their interests and self-concept as early as elementary school (Trice and McClellan, 1993; Maltese and Tai, 2009; van Tuijl and van der Molen, 2015; Lawson et al., 2018).

However, as mentioned before, most of the studies have focused on few subject domains and only certain aspects of motivational beliefs. Such a limitation means that more information is still needed especially with respect to understanding the domain-specific differentiation of motivational beliefs over time and its effects.

### Gendered difference in motivational beliefs and academic outcomes

The relationships between motivational beliefs and academic outcomes, as assumed by the SEVT model, are also influenced by gender. Gendered differences in domain-specific motivation are evident from findings discussed in numerous studies, with boys being inclined more toward mathematics and girls toward languages, and they tend to remain the same for early elementary school students to adolescents in secondary school (Jacobs et al., 2002; Watt, 2008, 2016; Frenzel et al., 2010; Nagy et al., 2010). In one example from a person-oriented study, Gaspard et al. (2019) confirmed the overrepresentation of a low math motivational profile for girls, coupled with high motivation in languages. A similar finding has also been presented, for example, in studies by Eccles and Wang (2015), Umarji et al. (2018), Jansen et al. (2021), Lazarides et al. (2021), and Oppermann et al. (2021). These studies show that gendered motivational beliefs (with more girls being motivated to study languages and boys to study more math-intensive subjects) are linked to differing domain-specific achievement and aspiration or choice of university major for girls and boys.

More specifically, Guo et al. (2018b) assessed how gender influences students’ personal *trajectories* with respect to the development of domain-specific motivational beliefs, which consequently shapes their occupational choices. Wang et al. (2013) also found that the main difference between the gender was influenced not only by the absolute levels of domain specific motivational beliefs but the different relative levels within the individual. Taken together, these studies stress the importance of intra-individual processes in the development of motivational beliefs, especially when considering the role of gender and its relation to academic outcomes.

To support younger students’ STEM engagement, it is therefore also important to further identify how the dynamics of motivational belief, both at different development stages and in relation to gender, influence consequent achievement and aspiration. The unique intra-individual differences have also demonstrated the importance of accounting for insights from person-oriented approaches when identifying the sub-population differences in the development of gendered motivational beliefs.

### The present study

In this study, we aim to extend current knowledge on the development of elementary school students’ motivational beliefs and their role in achievement and STEM aspiration. We analyze data from Finnish 5th and 6th grade students (around 11–12 years old) to understand the development of motivational beliefs during the late elementary school years. We collected data on students’ subjective task values and self-concept of ability in science, mathematics, and Finnish language to examine the effect of gender on the relationships involving motivational beliefs, achievement, and STEM aspiration. Specifically, we answer three research questions:

Research Question 1: (a) What motivational belief profiles can be identified from elementary school students in the domains of science, math, and Finnish language? (b) How stable are these profiles, and how likely are they to change from 5th to 6th grade?

We address the first question by analyzing and deriving motivational belief profiles for students in the 5th and 6th grades. Since other studies have already identified domain specific-profiles, such as math-specific and reading-specific profiles, in elementary school students (Nurmi and Aunola, 2005; Archambault et al., 2010; Viljaranta et al., 2016; Oppermann et al., 2021), we hypothesize that the domain-specific profiles are characterized by a clear differentiation in either science, math and/or Finnish. Additionally, following assumptions of DCT, we also hypothesize that levels of motivational beliefs should be similar in similar domains (i.e., science and math), and going opposite with dissimilar domains (i.e., math and/or science compared to language) (Hypothesis 1a). Moreover, since previous studies have also demonstrated the relative stability of these profiles, we also hypothesize that these domain-specific profiles are stable and consistent throughout 5th and 6th grade (Hypothesis 1b).

Research Question 2: To what extent are the motivational belief profiles associated with students’ achievement and STEM aspiration?

Students’ motivational profiles in science, math, and languages can predict academic outcomes with respect to achievement and STEM aspiration (Wigfield et al., 2009). Therefore, we expect to find a clear relationship between motivational belief in specific domains and achievement and STEM aspiration. Specifically, we assume that higher motivational belief in either math, science and/or Finnish will be reflected in higher achievement in the corresponding domain (Hypothesis 2a) and that higher motivational belief in math and science is also associated with higher STEM aspiration (Hypothesis 2b).

Research Question 3: To what extent do profiles, transitions, and their relation to students’ STEM aspirations and achievement differ based on gender?

To provide further evidence on the influence of gender on students’ motivation and academic outcomes, we focus on the relationship between the three. Previous studies based on the SEVT model have also discovered evidence of gendered differences in motivational profile membership, achievement, and STEM aspiration (Eccles, 2009; Wang and Degol, 2017; Wigfield and Eccles, 2020). Accordingly, we expect to find gender differences manifested in different ways. First, girls are overly represented in profile(s) identified with lower motivation in science and/or math and higher motivation in the Finnish language, and the profiles persist over time, while the profile memberships are the reverse for boys, but with similar persistence (Hypothesis 3a). Next, we also assume that girls—with lower science-math motivation levels—will have lower achievement scores in science and/or math, the opposite of boys (Hypothesis 3b). Girls with lower motivation in science-math will also have lower STEM aspirations, again the opposite of boys (Hypothesis 3c).

## Materials and methods

### Sample and procedure

Data from a Finnish longitudinal study (Name Removed for Reviewing Purpose) was used for this study, which followed students from seven schools in eastern Helsinki. The data collection process began in 2016 with first grade elementary school students at the age of seven or eight, and was always done each year in early February, which is in the middle of the school year. The development of their subject-specific motivation and aspirations were followed throughout the 6 years of elementary school.

In every data collection session, two researchers (or one assisted by a teacher) guided the students in answering the paper questionnaires. The researcher read each question and explained what each response means to the students (e.g., “one star in this one means I don’t like science at all.”). The assisting researcher or teacher walks around the class to check that every student can follow. During the data collection in year 5 and 6, either only one researcher is there or a teacher who have been trained administered the questionnaires as COVID pandemic situation limited the contact that is possible. The questionnaires were administered within one lesson (around 45 min) with short breaks in between.

The current study focused on students in their 5th and 6th grade (data collected in 2020 and 2021), and only students who participated in the data collection on those two waves are included. The final sample (*N* = 360, in 5th grade *N* girls = 200, boys = 160; in 6th grade, *N* girls = 192, boys = 164) had a mean age of 11.14 years (*SD* = 0.38) at 5th grade.

The study followed the ethical guidelines of the home institute. Parental consent was sought since participants in the study were elementary school-aged children. A description of the study and written permission forms were distributed to parents, and they had the opportunity to refuse to allow their child to participate in the study. Informed parental consent was obtained afterward for all the student participants. The headmasters and teachers from the participating schools were also informed about the study and agreed to the data collection schedule. Since the data collection was integrated with the students’ normal classroom activities, the class teacher organized separate activities for students who did not have permission to participate in the study. Permission to collect students’ data from schools was also obtained from the education division of the city of Helsinki (Kasko), with which we have cooperation agreements. According to the regulation from Kasko, no rewards or compensations are given for participants, either for students or the schools.

### Finnish education context

The Finnish compulsory education system consists of mandatory schooling for children aged 7–18 years. Throughout grades 1 to 6, or the lower classes of the comprehensive school, the students had lessons in, among other subjects, mathematics, Finnish, and science—labeled “environmental studies”—which is a combination of biology, geography, physics, chemistry, and health education (Oppetushallitus, 2014).

Students in grades 3–6 received at least 2–3 h of science or environmental studies, mathematics, and Finnish language lessons per week, which accumulate to approximately 10–18 lessons throughout the 3 years^1^. In science, the lessons focused on students’ knowledge and understanding, their research and working skills, and their values and attitudes toward the subject. In mathematics, the emphasis is on developing students’ mathematical thinking to be logical, precise, and creative. In Finnish language, students’ basic ability at listening, speaking, and reading is emphasized, while at the same time improving their self-expression, communication skills, and verbal awareness.

In terms of assessment, though students receive reports at the end of each school year, official national assessment criteria are only provided for students at the end of 6th grade to make sure the grades are comparable throughout the country. The grades ranged from from 4 to 10, with 5 as ‘Pass’ and 8 as ‘Good.’ The assessment criteria serves only as guidelines, and no national testing is conducted to determine different schooling tracks at this stage of schooling. Given this background, the Finnish context provided a unique opportunity to follow the development of elementary school students with less achievement-related feedback compared to some education systems in Europe (Hörner et al., 2015) such as in Germany or Austria, where students are streamed into different schooling tracks based on their achievements at the end of 4th grade.

### Measures

At both measurement points (grades 5 and 6), we used the same student-reported subjective task value and ability self-concept questionnaire based on a scale developed by Eccles et al. (1993). This assessment was done for each of the three domains (mathematics, science, and Finnish language). Students were also asked about their dream occupation or aspirations in the form of an open-ended question. As this is a self-reported questionnaire, at each measurement point the teacher and/or a researcher and an assistant would instruct and assist students to make sure they understood the questions, scales, and responses expected of them.

#### Subjective task value

We assessed students’ subjective task value with a Likert-type scale that ranged from 1 (“totally disagree”) to 5 (“totally agree”). The response choices were shown as stars of an increasing number and size, following the 1–5 range.

The students were asked to rate their intrinsic, utility, and attainment value in science, mathematics, and Finnish language domains. Intrinsic value was measured via three items: “I think the subject is fun”; “I like to do the schoolwork for this subject”; “I just like this subject.” Utility value was measured via another three items: “Knowledge of this subject helps me during my free time”; “Knowledge of this subject will be useful for me in my future profession”; “The subject is useful for me.” Finally, attainment value was also measured via three items: “I want to be good in (this subject)”; “I want to know a lot about this subject”; “This subject is important to me.”

For the analysis, the average student score for intrinsic, utility, and attainment value was calculated to represent the subjective task value for each subject. All scales had good reliability at each measurement time and in all domains (Time 1: Science: α = 0.88; Mathematics: α = 0.88; Finnish language: α = 0.87; Time 2: Science: α = 0.89; Mathematics: α = 0.88; Finnish language: α = 0.90).

#### Ability self-concept

Self-concept of ability was assessed with three items following the same Likert-type visual response format: “I am good in (this subject)”; “I am good at the schoolwork for this subject”; “The schoolwork for this subject is easy to me” (1 star = “totally disagree,” 5 stars = “totally agree”).

Again, an average score for each subject was calculated. The reliabilities were as follows: Time 1: Science: α = 0.83; Mathematics: α = 0.90; Finnish language: α = 0.84; Time 2: Science: α = 0.81; Mathematics: α = 0.90; Finnish language: α = 0.86.

#### Achievement

Students’ numerical grades for science, mathematics, and Finnish language were collected from the schools as a measure of student achievement. The grades are considered open information that is publicly accessible to all. Following Finnish school system mandates, a student is given a four as the lowest grade and a ten as the highest. On average, students in our sample have a mean of 8.414 (*SD* = 1.011) for science, 8.326 (*SD* = 1.134) for mathematics, and 8.354 (*SD* = 1.021) for Finnish language.

#### STEM aspiration

Students were asked an open-ended question about their dream jobs in both 5th and 6th grade, and their answers were combined to create a single aspiration variable and coded into occupational fields based on the ISCO-08 classifications (ILO, 2012). The encoding strictly followed the coding scheme. Based on these classifications, we derived two sets of coding schemes for the purpose of cross-validation. The *first coding scheme* included (a) mixed STEM fields (i.e., science and engineering professionals, health professionals, ICT professionals, science technicians, and associate professionals) and (b) non-STEM fields. The *second scheme* included (a) STEM-HBMS (health, biology, medical sciences), (b) STEM-MPCES (mathematics, physics, computer and engineering sciences), and (c) non-STEM fields.

### Missing value analysis and outliers

The sample for this study (*N* = 360, grades 5 and 6) represents 51.4% of the initial 700 students who were part of the longitudinal sample followed from grade 1. The main reasons for the high attrition rate were because students changed schools before 6th grade or dropped out of the study due to the ethics permission renewal process. The data collection permit and parental consent had to be renewed by 2015, and in the process fewer parents responded and/or gave their consent, resulting in a significant degree of attrition.

The final sample of 360 students also include missing data ranging from 0.3% (Finnish subjective task value in grade 5) to 49% (STEM aspiration in grade 6). The exact percentages of missing values are shown in Supplementary Table 1.

A comparison of the missing values for each study variable showed that gender was related to missing values in STEM aspiration, with boys having significantly more missing values in both years (*p* < 0.001 for grade 5, *p* = 0.009 for grade 6). Moreover, students with missing values in aspiration and score in each subject generally had a significantly lower science and Finnish language self-concept at grade 5. A full comparison of missingness is shown in Supplementary Table 5.

We also checked for possible outliers with z >/< 3.29, as suggested by Tabachnick et al. (2007), and found several potential multivariate outliers. These points were still included in the final analyses since they did not represent extreme values and did not affect the latent profile solutions.

### Statistical analysis steps

#### Preliminary analysis

First, we checked the basic correlations and dependency between the variables, then conducted a confirmatory factor analysis for both self-concept of ability and subjective task values for each subject and measurement time.

This was followed by a measurement invariance test to confirm invariance assumptions about factor loadings, item intercept(s), and variance across the domains and two time points, and we found empirical support for strict measurement invariance. The model fit results were satisfactory for all steps, with CFI and TLI values being close to 0.95, SRMR values being close to 0.08, and RMSEA values being close to 0.06 (Hu and Bentler, 1999), and a decrease of less than 0.010 in CFI values and 0.015 in RMSEA values during every step, evidence of measurement invariance as recommended by Chen (2007).

#### Latent profile analysis

Next, we explored latent profile solutions for each measurement point separately, as suggested in previous longitudinal person-oriented study (Tang et al., 2021) and based on recommendations by Spurk et al. (2020). All models were estimated using Mplus 8.6 (Muthén and Muthén, 1998-2017) using a robust maximum likelihood estimator with the assistance of the R package MplusAutomation (Hallquist and Wiley, 2018).

We estimated up to six profiles using composite subjective task values and self-concept of ability for each subject (i.e., science, mathematics, and Finnish language) by freely estimating the means of these indicators. In terms of correlation and variances, we used the default model specification from Mplus: all variables are uncorrelated with all variables within the class and equal variances. To decide on the final number of profiles, we relied on both theoretical considerations by examining the difference in the mean for each profile and checking the fit information criteria. In this study, we relied on the Akaike Information Criterion (AIC), Consistent AIC (CAIC), Bayesian Information Criterion (BIC), and adjusted Bayesian Information Criterion (ABIC). Lower values for the four information criteria indicate a more optimal number of profile solutions. Visualization of the fit using elbow plots was used to compare the information criteria. The plot aided in deciding on the optimal solution by showing the number of profile solutions at which the slope started to flatten.

#### Latent transition analysis

After selecting the optimal number of profile solutions, we tested profile similarity by integrating the solutions from the two time points into a longitudinal latent profile analysis (LPA) model following the steps described by Morin and Litalien (2017). Four steps were followed: (1) *configural similarity* was tested to check if the numbers of profiles remained the same over time, using the same indicators with no constraints; (2) *structural similarity* was verified by constraining the indicator intercepts to note any similarities in global shape over time; (3) *dispersion similarity* was tested by constraining indicator variances over time to check the stability of within-profile variability; (4) *distributional similarity* was the final test, done by further constraining profile probabilities over time to confirm the stability of each profile’s relative size. For each of these steps, two of the CAIC, BIC, and ABIC values should be lower compared to the last model to show evidence that the assumption is correct (Ryoo et al., 2018). After confirming the most similar model, we then converted it into a longitudinal latent transition analysis (LTA) model to identify stability and changes across latent profile membership over time.

#### Regression with predictors and outcomes

We used the final LTA model to test the extent to which students’ profile membership and transition were related to their educational achievement and STEM aspiration. To examine the gender difference in profile membership, we used the three-step approach (R3STEP) in Mplus, as described by Asparouhov and Muthén (2014). We modeled gender as the predictor of latent profile membership through logistic regression. To evaluate gender difference in transition probabilities, we next used the KNOWNCLASS function. Finally, to test the extent to which gender and profile membership are related to students’ outcomes, we applied a manual auxiliary three-step approach with a distal outcome (Asparouhov and Muthén, 2014). We used both gender and latent profiles as predictors, while treating the latent profiles as the auxiliary variable and regressing them based on students’ grades (in science, math, and Finnish language) and STEM aspirations (with both coding schemes) as the outcome variable.

## Results

### Descriptive analysis

Means and zero-order correlations of the variables included in the analyses are reported in Supplementary Table 1 for each motivational belief. The highest means for both self-concept and task values were in mathematics for students in both 5th and 6th grade. Mean comparisons showed a slight decrease in motivational beliefs in all subjects except for science self-concept. The results of the chi-square test of independence for the relationship between gender and STEM aspiration were significant, χ^2^(1, *N* = 360) = 11.205, *p* < 0.00, indicating a dependency between gender and aspiration (Supplementary Table 2). Measurement invariance testing for grades 5 and 6 showed that strict invariance of loadings, intercepts, and residual uniqueness was achieved for all three domain-specific self-concept and subject task value (STV) measures (see Supplementary Table 3 for details of the fit summary).

### Latent profiles of motivational belief and profile transition

The final profile solution was chosen based on theoretical meaningfulness, statistical criteria, and interpretability. The cross-sectional LPA for both time points suggested that the fit indices continued to improve as each profile was added, with lower AIC, CAIC, BIC, and ABIC values. However, the elbow plots at both time points showed that these fit indices started dropping less after the fourth profile, and profiles representing less than 5% of the participants emerged starting with the five-profile solution (Supplementary Figure 1 and Supplementary Table 4). Therefore, after considering the fit indices, interpretation, and the meaningful distinction of the added profiles, we chose the four-profile solution for both time points.

**FIGURE 1:**
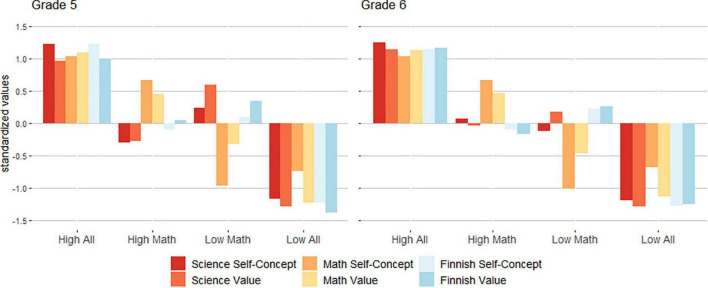
Profiles in both years.

Following this step, we employed a longitudinal LPA model to test similarities in the four-profile solution at both time points. Partial distributional similarity was retained with the lowest BIC and SABIC values. This implies that the number of profiles, intercept, variance, and group size were similar over time. We used this model for all further analyses. The profiles derived from this final model are illustrated in Figure 1.

With this four-profile solution, we labeled the first profile *high all* (grade 5 = 27.2%, *n* = 98; grade 6 = 15.3%, *n* = 55), as students in this profile showed high motivation in all domains. The second profile, with the most students, we labeled *high mathematics* (grade 5 = 51.4%, *n* = 185; grade 6 = 41.7%, *n* = 150), and it describes students with a moderate level of motivation in science and Finnish language and a high level of motivation in mathematics. In the third profile, *low mathematics* (grade 5 = 12.8%, *n* = 46; grade 6 = 9.4%, *n* = 34), students also exhibited a moderate level of motivation in science and Finnish language and low motivation in mathematics. The final and smallest profile, *low all* (grade 5 = 8.6%, *n* = 31; grade 6 = 7.2%, *n* = 26), describes students who reported low motivation in all subjects.

Following the four-profile solution, LTA provided the transition probabilities of each profile for students in the 5th and 6th grades. The probabilities are reported in Table 1. Students in the hi*gh mathematics* profile exhibited the greatest stability (89%) followed by those in the l*ow mathematics* profile (78%). Some students also transitioned both to the math-specific profiles and to more general profiles. The highest rate of transition was observed for students moving from the *high all* to *high mathematics* profile (19%), followed by students moving from the *low mathematics* to *low all* profile, and vice versa (17% for each), and those moving from the *low all* to the *high all* profile (15%).

**TABLE 1:** Transition probabilities.

	Transition probabilities to 6th grade profiles			
*Profiles at 5th grade*	*High all*	*High math*	*Low math*	*Low all*
***High all***	**0.729**	0.191	0.074	0.006
***High math***	0.000	**0.894**	0.000	0.106
***Low math***	0.016	0.033	**0.781**	0.170
***Low all***	0.154	0.046	0.170	**0.630**
*(Girls)*				
***High all***	**0.746**	0.090	0.130	0.033
***High math***	0.000	**0.999**	0.000	0.001
***Low math***	0.026	0.001	**0.753**	0.220
***Low all***	0.140	0.047	0.271	**0.542**
*(Boys)*				
***High all***	**0.709**	0.268	0.024	0.000
***High math***	0.003	**0.790**	0.002	0.205
***Low math***	0.000	0.380	**0.620**	0.000
***Low all***	0.159	0.041	0.103	**0.696**

The values in bold represents profile stability from 5th to 6th grade.

### Latent profile membership, achievement, and aspiration

With respect to grades, regression analyses found that students’ membership in math-specific profiles (i.e., *high mathematics* or *low mathematics*) are associated with differences in their levels of math achievement, as depicted in Table 2. Students in the *high mathematics* profile had similar math grades compared to students in the *high all* profile, although this finding cannot be generalized in the same way for science and Finnish language grades. We also detected a similar pattern when comparing students in the *low mathematics* and *low all* profiles, as they had similar math grades, but those in the former profile also had significantly higher science and Finnish language grades than students in the latter profile.

**TABLE 2:** Achievement difference in each profile.

	High all (P1)	High math (P2)	Low math (P3)	Low all (P4)	Summary of significant differences
*Science*	9.016 [8.819;9.214]	8.653 [8.496; 8.811]	8.265 [8.035; 8.495]	7.784 [7.474; 8.094]	P1 > P2 > P3 > P4
*Math*	9.039 [8.854; 9.225]	8.828 [8.697; 8.960]	7.378 [7.062; 7.693]	7.613 [7.120; 8.106]	(P1 = P2) > (P3 = P4)
*Finnish*	8.929 [8.752; 9.105]	8.571 [8.373; 8.769]	8.309 [8.052; 8.567]	7.673 [7.253; 8.092]	P1 > (P2 = P3) > P4

Grades in Finnish schools are expressed in a 4-10 range; 4 as the lowest grade, 10 the highest.

We also tested the association of the profiles with STEM aspiration, which revealed a different pattern compared to their achievement levels (Table 3). We found no STEM aspiration difference based on the math-specific profiles, unlike the achievement pattern, with only students in the *low all* profile showing significantly less STEM aspiration than those in all the other profiles. We noted no further difference between the other three profiles, as students in the *high all*, *high mathematics* and *low mathematics* profiles exhibited comparable levels of STEM aspiration. The patterns of the results are the same for both STEM aspiration coded for only STEM (mix) and those coded for HBMS and MPCES fields.

**TABLE 3:** Science, technology, engineering and math (STEM) aspiration difference in each profile.

	High all (P1)	High math (P2)	Low math (P3)	Low all (P4)	Summary of significant differences
*STEM Aspiration*	0.406 [0.288; 0.523]	0.379 [0.271; 0.487]	0.254 [0.123; 0.384]	0.083 [0.029; 0.136]	P1 = P2 = P3 > P4
*HBMS-MPCES Aspiration*	0.566 [0.364; 0.769]	0.527 [0.349; 0.706]	0.297 [0.153; 0.440]	0.115 [0.054; 0.176]	P1 = P2 = P3 > P4

Aspiration was coded in two ways: 0 = Non-STEM, 1 = STEM; or 0 = Non-STEM, 1 = HBMS (Health, Bio and Medical Science), 2 = MPCES (Math, Physics, Computer and Engineering Sciences).

### Gendered profile membership and transition

At both time points, we found gender differences in *low mathematics* profile, with more girls exhibiting moderate motivation and placed in this profile (78%; *n* = 36 in grade 5 and 79%; *n* = 27 in grade 6). This contrasts with the approximately equal distribution of boys and girls in all the other profiles (proportion of girls: ∼50% in *high all*, ∼54% in *high mathematics*, ∼56% in *low all*. See Supplementary Table 4). Logistic regression analysis showed that girls have a higher likelihood of being placed in this profile compared to other profiles, as described in Table 4.

**TABLE 4:** Gendered difference in profile membership.

	Low math				High math				High all			
	*B*	*SE*	*p*	*OR*	*B*	*SE*	*p*	*OR*	*B*	*SE*	*P*	*OR*
*Ref: Low all*												
Female	–1.258	0.516	0.015	0.284	0.053	0.382	0.890	1.054	0.101	0.374	0.786	1.107
*Ref: Low math*												
Female					1.311	0.458	0.004	3.709	1.360	0.412	0.001	3.894
*Ref: High math*												
Female									0.049	0.295	0.869	1.050

In terms of transition, adding KNOWNCLASS to the model showed that girls especially exhibit higher levels of stability in the mathematic-specific profiles, as described in the lower part of Table 1. Girls have a 99% probability of remaining in the hi*gh mathematics* profile (compared to 79% for boys) and a 75% probability of remaining in the lo*w mathematics* profile (compared to 62% for boys). Moreover, more boys seem to transition to the h*igh mathematics* profile (26% from h*igh all*, 38% from l*ow mathematics*, and 4% from l*ow all*) compared to girls, who exhibited a less than 10% transition probability from all the other profiles combined.

### Gendered profiles, achievement, and aspiration

We found gendered differences in student academic performance at grade 6 within the different profiles. As described in Table 5, regression analysis revealed differences between girls and boys in the *high mathematics*, *low mathematics*, and *low all* profiles. With respect to students in the *high mathematics* profile, girls achieve significantly higher in science and Finnish language, but not in math. With respect to students in the *low mathematics* and *low all* profiles, girls also achieve significantly higher in Finnish language compared to boys.

**TABLE 5:** Gender effect on outcomes within profiles.

	High all			High math			Low math			Low all		
	*B*	*SE*	*p*	*B*	*SE*	*p*	*B*	*SE*	*p*	*B*	*SE*	*p*
*Achievement: Science*	–0.143	0.232	0.536	–**0.437**	**0.192**	**0.023**	–0.367	0.255	0.151	–0.384	0.358	0.284
*Achievement: Math*	0.062	0.222	0.781	0.169	0.157	0.280	–0.515	0.315	0.102	–0.147	0.613	0.811
*Achievement: Finnish*	–0.287	0.203	0.157	–**0.680**	**0.201**	**0.001**	–**1.022**	**0.290**	**0.000**	–**0.951**	**0.447**	**0.033**
*STEM aspiration*	–**0.298**	**0.142**	**0.036**	0.015	0.138	0.915	–0.109	0.144	0.450	–0.028	0.060	0.640
*HBMS-MPCES aspiration*	–0.419	0.362	0.247	0.415	0.787	0.598	–0.231	0.183	0.208	–0.094	0.081	0.248

HBMS, Health, Bio and Medical Science; MPCES, Math, Physics, Computer and Engineering Sciences; Girls coded as 1; Boys 2. Significantly different profile are highlighted in bold.

In terms of STEM aspiration, presented in the lower part of Table 5, the differences between girls and boys within the profiles are not as visible. We found that girls in the *high all* profile have significantly more interest in aspiring to a STEM career. Otherwise, we detected no differences between girls and boys in terms of their STEM aspiration. Likewise, we found no difference when coding STEM for HBMS and MPCES. In other words, we observed no differences between girls and boys within each profile in terms of their aspiring to an HBMS, MPCES, or non-STEM occupation.

## Discussion

Our study examines the influence of gender on motivational belief patterns and students’ academic outcomes at the end of elementary school. Guided by expectancy value theory (Eccles et al., 1983; Eccles and Wigfield, 2020) and dimensional comparison theory (Moller and Marsh, 2013), we analyzed students’ motivational patterns in specific domains and connected them to their levels of achievement and STEM aspirations. Our findings provide clear evidence of gender differences in students’ motivational profiles, even among elementary school students, and the different profiles are associated with their achievement levels and aspirations.

### Domain-specific motivational profiles and their stability

Our first aim was to identify intra-individual motivational patterns among students in the 5th and 6th grades. As a result of latent profile analysis, we identified four different motivational belief profiles throughout the 2 years (Figure 1). Two of the profiles were characterized by moderate motivation levels in science and Finnish language, one with a high motivation level in math (*high mathematics*), and the other profile with a low motivation level in math (*low mathematics*). The remaining two profiles showed a general pattern in all three domains (science, math, and Finnish language), one with high motivation levels for all domains (*high all*), and the other with low motivation levels (*low all*).

The first major finding partially supports our first hypothesis (1a) regarding a clear domain-specific differentiation in motivational belief profiles. The four profiles, two of which were characterized by strong motivation in math, confirmed that students have formed some domain-specific motivational beliefs already by 5th and 6th grade. This finding is similar to what had been reported in other person-oriented studies, such as studies by Nurmi and Aunola (2005); Viljaranta et al. (2016), and Oppermann et al. (2021). They also found that elementary school students have already developed clear intrinsic value and self-concept of ability toward mathematics. Our findings, however, add a new piece of evidence to the existing literature since we conceptualized motivational belief through task value perspective. By measuring subjective task value as a composite of intrinsic, attainment, and utility values (together with self-concept of ability), we still found that a clear domain-specific motivational profile for math has developed among students at this age.

On the other hand, we observed a lack of a specific motivational profile dedicated to science and/or language, contrary to the profiles found among older students. Past studies conducted using data collected from adolescents, such as those by Gaspard et al. (2018, 2019), Jansen et al. (2021), and Lazarides et al. (2021) identified profiles characterized by specific science and/or language motivation, not only specific profiles for mathematics. The difference between the results may be explained by the fact that the prior studies focused on older students, at which point students have developed more stable motivational profiles (Lazarides et al., 2016, 2019).

Additionally, the levels of motivational beliefs in the math-specific profiles suggested that the assumed *similar domains* (i.e., science and math) were growing in opposite directions, and what we assume as *dissimilar* (science and Finnish language) had similar levels of motivation. These profiles that we observed suggest that elementary school students have not developed the ability to distinguish the similarity and dissimilarity between the domains, particularly in science, as much as adolescents.

One of the reasons is that in the Finnish elementary school system the domain “science” is a mix of different subjects (i.e., biology, geography, physics, chemistry, and health education). Such a context most likely leads to less specialization in elementary school students since they perceive “science” less concretely. In other words, students at the Finnish elementary school stage have not been exposed to the differences between specific science domains (e.g., physics versus biology) or between the science domain and more language-intensive domains. After further exposure, older students could develop more domain-specific motivational profiles, as demonstrated by other person-oriented studies focusing on Finnish adolescents (Chow and Salmela-Aro, 2011; Guo et al., 2018b). We can, therefore, assume that further differentiation of domain-specific motivational profiles takes place later in students’ development, as they become more exposed to the differences between domains, and not yet when they are in elementary school. This finding also resonates with the SEVT model (Eccles and Wigfield, 2020), which suggests that values are situationally bounded. A recent study also demonstrated that expectancy and task values are situative across domains, grade levels, and countries (Tang et al., 2022).

With regards to the transition between 5th and 6th grade, we found generally stable profile memberships, confirming hypothesis 1b, with only a few students shifting to different profiles. As suggested by the high odds of remaining in the same profile (above 60% for all profiles), students’ general motivational level did not change during these years. This is true especially for students with high motivation. Some students did move to the more specialized *high mathematics* profile from the more general *high all* profile (around 19%), but generally they remained highly motivated. We noted a similar stable motivational trend for students with lower motivation, although with lower levels of stability.

The less stable profiles hint at the fact that students with lower motivation levels can still be pushed and encouraged to do better. Although 17% of students in either the *low all* or *low mathematics* profile remained in the low motivation profiles, 15% of students who were in the *low all* profile during 5th grade moved to the *high all* profile in 6th grade. This finding is encouraging in contrast to the more general trend that students will only continue to exhibit lower competence beliefs and task values as they grow older (Archambault et al., 2010; Musu-Gillette et al., 2015; Gaspard et al., 2017). During late elementary school years, some changes are still occurring and students also still develop an upward trajectory of motivation and not only a declining trajectory.

### Domain-specific motivational profiles, achievement, and STEM aspiration

Our next major finding is that the domain-specific motivational profile is closely related to student achievement, as we expected for hypothesis 2a. We found that students fitting profile(s) with higher motivation levels in math tended to achieve better scores in the same domain. We also found the opposite effect to be true with respect to those students with low motivation levels in math. This finding is not as clearly demonstrated for the other two subjects, as we discovered no specific profiles identified with only a science or language focus. However, the general trend remained the same and confirmed our hypothesis: students in higher motivation profiles exhibited significantly higher achievement.

This finding confirms the direction of the relationship between domain-specific motivation and achievement that other studies have reported before. Even in elementary school students, the higher math motivation profile is associated with higher achievement or performance in this domain (Nurmi and Aunola, 2005; Viljaranta et al., 2016). This relationship most likely is a result of deeper engagement and persistent learning in domains where students already exhibit higher motivation (Wang et al., 2013; Tang et al., 2019).

In relation to aspiration, we found that the students’ motivational profiles provide only limited information on their STEM aspiration. In this study, the *low all* profile was the only profile that successfully predicted lower STEM aspirations compared to the other profiles. Otherwise, we found no significant difference between all the other profiles in term of the students’ STEM aspirations. More interestingly, although we identified profiles with a math-specific motivation, such motivation is not connected strongly to differences in STEM aspirations.

The limited association between domain-specific profile membership and STEM aspiration differs slightly from results presented in previous studies. Past studies have shown that early elementary school students characterized by higher math and science task values are more likely to have greater STEM aspirations (Vinni-Laakso et al., 2019; Oppermann et al., 2021). Studies done among older students have also found that those with higher math and science-specific motivation levels have greater aspiration to pursue a career in physical science or information technology (Guo et al., 2018b; Lazarides et al., 2021) and are the ones who typically end up choosing a STEM-related career (Wang and Degol, 2013; Wang et al., 2015; Guo et al., 2018a; Gaspard et al., 2019). In comparison, we did not find support for a strong association between membership in a math-specific profile and greater STEM aspirations.

The lack of evidence for such a strong association may suggest that students’ STEM aspirations do not necessarily rely only on their math-specific motivation levels. A common finding for all the profiles showing comparable STEM aspiration (i.e., *high all*, *high mathematics*, *low mathematics*) is that they contained students with high and/or moderate science motivation levels. We can assume, therefore, that science motivation, in addition to math motivation, can also act as a source of interest contributing to strong STEM aspirations, even when it is not yet as well differentiated during elementary school. This assumption is also consistent when we consider the fact that students in the *low all* profile tend to have low motivation in all domains, leaving them with no buffer to even entertain the idea of aspiring to a STEM-related career path.

Taken together, even though elementary school students only show differentiation in terms of math-specific motivation levels, higher motivation in math and science is still associated with a greater likelihood of aspiring to a STEM-related career. With further differentiation, as described among older students, a clearer association between domain-specific motivation (in math and/or science) and STEM aspiration is more typically observed.

For elementary school students, we can only identify a clear distinction in math-specific profiles, even though we also considered other domains and all task value constructs. The profiles thus have only a limited relationship with STEM aspiration. This finding implies that strong coupling between clear subject-specific motivational beliefs and STEM aspiration has not taken place among elementary school students.

### Gendered differences in motivational profiles

As we had expected with respect to hypothesis 3a, there are gendered differences in the motivational profile membership and the extent to which students transition between them. The logistic regression result showed that significantly more girls are in the *low mathematics* profile compared to other profiles. In terms of mathematics, we also discovered a difference in transition probabilities for both genders, with boys being more likely than girls to shift to the *high mathematics* profile in grade 6. These findings imply that indeed for students in their final years of elementary school, we can already observe gendered domain-specific motivational differences, especially in mathematics.

The gender differences among motivational profiles align with findings from previous studies. Person-oriented studies of older students have also shown that girls predominate in profiles characterized by a low level of motivation in mathematics (Chow and Salmela-Aro, 2011; Chow et al., 2012; Gaspard et al., 2019). This runs parallel with boys developing a higher math self-concept and greater self-confidence (Frenzel et al., 2010; Nagy et al., 2010). Longitudinally, Guo et al. (2018b) found that starting from grade 9, Finnish students tend to develop along different gendered motivational trajectories for different domains. Girls tend to place greater value on Finnish language and social subjects and less value on math and science. Exhibiting an opposite trend, boys place more value on math and science in their later school years.

The similar trend of motivational differences between girls and boys should serve as a warning of the risks to both genders. The transition odds for girls with low math motivation suggest that they will most likely continue to lose motivation for math, or even generally move more in the direction of lower general motivation. In other words, girls with low math motivation might be stuck in a vicious cycle of losing motivation in other subjects over time. This trend is not the same for boys: regardless of whether they have a higher or lower motivation in math in 5th or 6th grade, the odds are greater that they will end up in the higher motivational profiles in later years. It is important, therefore, to address the possibility that girls are at greater risk of continuing to lose motivation, especially in math, which will influence other outcomes as well.

### Gendered motivational profiles and achievement

With respect to hypothesis (3b), we found that girls and boys achieved differently within certain profiles. In the *high mathematics* profile, the girls had significantly higher science and Finnish languages grades than the boys. The achievement gap also proved significant for the *low mathematics* and *low all* profiles, with girls having significantly higher Finnish language grades. Aside from the differences in science and Finnish language, we noted no gender differences in mathematic achievement within the profiles. In sum, it is worth noting that during these elementary school years, girls generally have higher achievement scores in science and Finnish language compared to boys.

It is interesting to note that we did not find evidence of gender difference in math grades within the profiles. This suggests that different math-specific motivation levels, as represented by the profiles, can sufficiently explain the differences in math achievement among students. Considering the fact that we also found gendered differences in the profile membership, this finding provides further evidence that gender influences students’ outcomes through differences in math motivation. Consistent with SEVT, this result suggests that even among elementary school students, the influence of gender on domain-specific achievement is connected significantly with its influence on domain-specific motivation levels.

Moreover, with respect to DCT our findings indicate the possible start of a divergence in students’ motivation levels and outcomes in 5th and 6th grades. According to the theory, one of the ways in which students are motivated to study a subject is through evaluating their performance in similar and dissimilar domains. Our results show that some girls who are equally as motivated in mathematics as boys still have higher achievement scores in science and Finnish languages. Based on the theory, the situation likely suggests that those girls will ultimately transition away from the math-intensive domain as they notice their strong performance in other dissimilar domains, such as in Finnish language.

Furthermore, in addition to processes related to domain-specific motivation, gender might also influence achievement through other means. Our results confirm that girls perform better in science and Finnish language, a finding confirmed by other studies as well (Wang and Degol, 2013; Keller et al., 2021), although we did not observe differences in the motivational profiles specifically for those domains. This indicates that gender also influences achievement through other processes beyond just domain-specific motivation, such as through different socialization processes (Eccles, 2009) and stereotypes (Miller et al., 2018; Master et al., 2021).

### Gendered motivational profiles and science, technology, engineering and math aspiration

In contrast to hypothesis 3c, we did not find clear gender differences in STEM aspiration within domain-specific motivational profiles. The regression result showed no significant gender difference except for those within the *high all* profile, where more girls aspire to a STEM-related career. This result suggests that when the students are highly motivated in general, girls have higher STEM aspirations than boys. However, this gendered difference disappeared when we regressed STEM aspiration for HBMS and MPCES.

When taking this result into consideration along with the other findings, this study provides further understanding of gendered differences in association with motivation and STEM aspiration. The regression with gender provided evidence that only girls in the *high all* profile have significantly higher STEM aspirations. Based on these findings, we can assume that that low general motivation in elementary school students rather than higher levels of motivation makes a more significant difference in their STEM aspirations. However, if we are observing gender difference, it is only for the most part visible in more highly motivated students.

Another interesting point to note is that the result of the regression singled out highly motivated and high achieving girls as those having higher aspirations to pursue a STEM-related career. This result aligns with Finnish statistics of university students, which records that only around 25–30% of students enrolled are women in Information and Communication Technologies (ICT) or Engineering, Manufacturing and Construction, a contrast with Health and Welfare fields, in which 70% of students are women (StatisticsFinland, 2022). In other words, this finding seems to support the idea that high achieving girls, when they choose to enter STEM fields, are more likely to choose HBMS fields compared to MPCES.

On the other hand, this result is in contrast with our expectation that girls are the ones with less motivation to try hard in math and science, thus having less interest in pursuing a STEM-related career. The contradictory result in terms of more girls aspiring to a STEM-related career compared to boys may point to the tendency for girls to have a wider range of interests, higher levels of achievement, and therefore, more aspirational choice (Wang and Degol, 2013).

A note of caution is due here regarding our interpretations since the aspiration variable had the lowest response rate. A large proportion (49%) of the students did not answer the question about their dream job or they responded that they are unsure of their aspirations. In other words, a lack of awareness about possible career choices among students at this stage might be one possible reason for the lack of aspirational difference we observed.

Nevertheless, our findings still show that clear gendered differences with respect to higher STEM aspiration can only be detected among highly motivated students, which is slightly different from results presented in earlier studies. For instance, Guo et al. (2018b) found that throughout adolescence, girls have the tendency to exhibit decreasing levels of task values toward math and science, which is strongly associated with lower participation in STEM career. This finding also accords with a study by Oppermann et al. (2021) that focused on elementary school students’ intrinsic value and self-concept in different domains. They found a similar result that stable math-specific motivation levels in elementary school students can mostly be observed in boys, and the motivation pattern was strongly associated with higher aspiration levels toward STEM.

The discrepancy between previous findings and our results may indicate that the development of students’ STEM aspiration in late elementary school is not as straightforward. We should consider how much elementary school students understand and perceive different subject domains, whether similar or dissimilar ones, and different motivational constructs. According to our findings, most students did not have a well-developed and stable means of clearly differentiating between domain-specific motivation at this late elementary school stage, which is different than adolescents (Muenks et al., 2018; Wan et al., 2021). This developmental difference most likely explains the less clear associations between students’ gendered motivation levels and outcomes, including their STEM aspirations. Moreover, as we argued in the previous section, students most likely have developed their aspiration at this point based on more information, such as certain stereotypes about which career is suitable for which gender (Chambers et al., 2018; Miller et al., 2018). However, this process might not yet necessarily manifest itself in the relationship mediated by motivation in 5*^th^* and 6*^th^* grade, and it is still likely that students from different profiles develop different STEM aspiration as they grow older (Mello, 2008; Lawson et al., 2018).

### Implications of research

First, we found evidence that students’ domain-specific differentiation takes place at the end of elementary school, even though it is not as well-differentiated. This finding supports the theoretical assumption regarding the developmental difference described by SEVT and the personal domain comparison processes described by DCT. Moreover, this finding also should support practices by educators. Understanding how students are developing different personal motivational beliefs for different domains at this stage should inform teachers’ instructional processes.

Next, our findings also suggested the need to critically consider the possible trajectories for students’ further motivational development and its impact on students’ academic outcomes. We found an association between levels of math-specific motivation and achievement, which suggests the need for educators to pay specific attention to students’ domain-specific motivation in supporting their achievement. Furthermore, we also observed less motivated students who transitioned to higher motivation profiles, hinting that there are still possibilities for change. Perhaps more possibilities exist for students to increase their motivational development in relation with achievement, which is a promising insight.

We also found evidence of gendered differences in the motivational profile membership, transitioning to higher motivation profiles, and academic outcomes. Significantly more girls in our study displayed low math motivation, while more boys transitioned to higher math motivation profiles in 6th grade. These gendered tendencies were significantly related to those particular students’ achievement levels, but not to their STEM aspirations. Therefore, critical attention is needed to address the motivation levels and outcomes among students in both genders.

In terms of the gendered achievement gap, specific attention is needed for girls with lower motivation levels. Our results suggest that many girls already show low math motivation and a greater tendency to have even lower general motivation. Addressing this issue as early as possible is critical for such students, as it is more important for them not to continue dropping in their motivation and achievement levels, thus preventing the continuation of a vicious cycle.

On the other hand, our results also suggest critical points to be addressed further in terms of enhancing aspiration and interest in STEM. The evidence we found suggests that in this age group, gendered motivation is still quite malleable and is not reflected clearly in students’ aspiration levels. For instance, we found girls who still *have* a more open attitude toward math continue to perform well in this domain, and girls who have equal, if not higher, academic performance in all domains compared to boys, even when they have similar levels of motivation. Some of the more highly motivated girls even showed greater interest in STEM. However, previous studies also found that they are also the students most likely to be steered away from such choices as their value hierarchy is increasingly influenced by their broader achievements and interests (Wang et al., 2013).

It is therefore important to to develop further studies addressing the dynamics of factors related to why girls end up not pursuing STEM careers, especially in MPCES, despite their high motivation and achievement. Especially in the Finnish context – understanding the dynamics between early subjective STEM experiences and social and/or environmental barriers is necessary (Schoon, 2001).

Furthermore, it is also critical to help students stay motivated and encourage them to consider a STEM-related field for future studies and/or work through designing interventions for students at this stage. For example, as we found that students seem to not base their aspiration on their domain-specific interests, development of interventions aimed at exposing them to examples and possibilities to not only cultivate their STEM interests but also STEM career aspiration (e.g., from exposing them to different role models and narratives as suggested by Luttenberger et al. (2019). Additionally, as our findings also hints at potential effects of stereotypes and socialization effects in STEM interest development, interventions designed to challenge STEM-stereotypes that students might have developed is necessary. Most importantly, activities and programs that support students’ STEM interest in terms of understanding a wider STEM relevance at school (e.g., as described by Harackiewicz et al., 2014; Gaspard et al., 2015) should be of prime importance to maintain students’ STEM motivation.

### Limitations and further research

Our study provides further insight into the relationship between gender, motivational beliefs, and their longitudinal effect on students’ achievement and STEM aspirations at the end of elementary school. However, certain limitations need to be considered. First, in terms of statistical power, our sample was not large enough to provide further details on different associations. For example, with the current sample size we could not test the relationship between the transition within 2 years and the students’ academic outcomes. We also could not officially test the association between the profiles with specific STEM aspiration (HBMS-MPCES) since our sample was not large enough compared to the very limited response rate from students.

Second, we only used data from two time points (in Grade 5 and 6) to assess the students’ motivational development, with achievement data only for grade 6. These limitations do not allow a complete insight into students’ overall motivational development in elementary school, especially in relationship with a key outcome, such as achievement. Thus, future studies should consider a longer time span to provide more nuanced understanding of students’ longitudinal motivation development and its relationship with key outcomes.

Finally, we derived most variables from self-report measures, except for the students’ grades. Further studies that also focus on the influence of different factors in shaping students’ academic outcomes would need to consider more sources of information.

## Conclusion

This study provides evidence that students in the last years of elementary school have developed different motivational profiles associated with their academic outcomes. We identified four different motivational profiles, two of which were specific to math but none specific to science or the verbal domain. This domain specialization remains stable throughout the school years and is most likely only enhanced as students grow older. We identified an association between higher math motivation levels and higher grades in each of the profiles. On the other hand, math-specific profile membership was not strongly connected to higher STEM aspiration, although general low motivation is associated with lower STEM aspiration. Taken together, our findings provide evidence that domain comparison processes are indeed already underway even among elementary school students, and that different profile membership influences students’ achievement and aspiration in different ways.

We also provided more insight on the relationship between critical outcomes, such as students’ grades and STEM aspiration, and their motivational beliefs and gender. Girls and boys showed different tendencies for profile membership and transition. In general, girls are showing significantly lower motivation in math and lower transition toward higher motivation in math. This gendered tendency was clearly reflected in their outcome, such as their math achievement scores. On the other hand, girls showed higher achievement in science and Finnish compared to boys with similar motivation, and some girls with high motivation even showed higher STEM aspiration. These findings present the different opportunities and risks for their development that requires further exploration.

In sum, to support academic outcomes for both girls and boys it is important to consider their gendered motivational beliefs. Understanding these associations is important in light of supporting students’ development along different career pathways, and future studies can and should build upon these findings to further identify critical periods and constructs for intervention and improvement.

## Data availability statement

The data analyzed in this study was subject to the following licenses/restrictions: this study used an already existing dataset that includes sensitive data (pseudonymized dataset linked to personal identity of students). The raw dataset will only be shared publicly according to the regulations from Academy of Finland scheme by the PIs. Queries regarding these datasets should be directed to the corresponding authors KO, kezia.olive@helsinki.fi or XT, tangxin09@gmail.com.

## Ethics statement

Ethical review and approval was not required for the study on human participants in accordance with the local legislation and institutional requirements. Written informed consent to participate in this study was provided by the participants’ legal guardian/next of kin.

## Author contributions

KO performed the statistical analysis together with XT. KO wrote the manuscript with suggestions and evaluations from XT and KS-A. XT and KS-A co-supervised the whole project. KJ and AL collected the data and provided extensive context information. All authors discussed the results and contributed to the final manuscript.

## Footnotes

1
https://www.oph.fi/sites/default/files/documents/distribution-of-lesson-hours-in-basic-education-2020.pdf
