# Supplementary material for: Gendered difference in motivational profiles, achievement, and STEM aspiration of elementary school students
Source: Front Psychol. 2022 Aug 29;13:954325. doi: 10.3389/fpsyg.2022.954325 (PMC9469012; doi:10.3389/fpsyg.2022.954325)
Supplement: Supplementary file 1 [file Data_Sheet_1.docx]

Supplementary Material

# Supplementary Figures and Tables

## Supplementary Figures


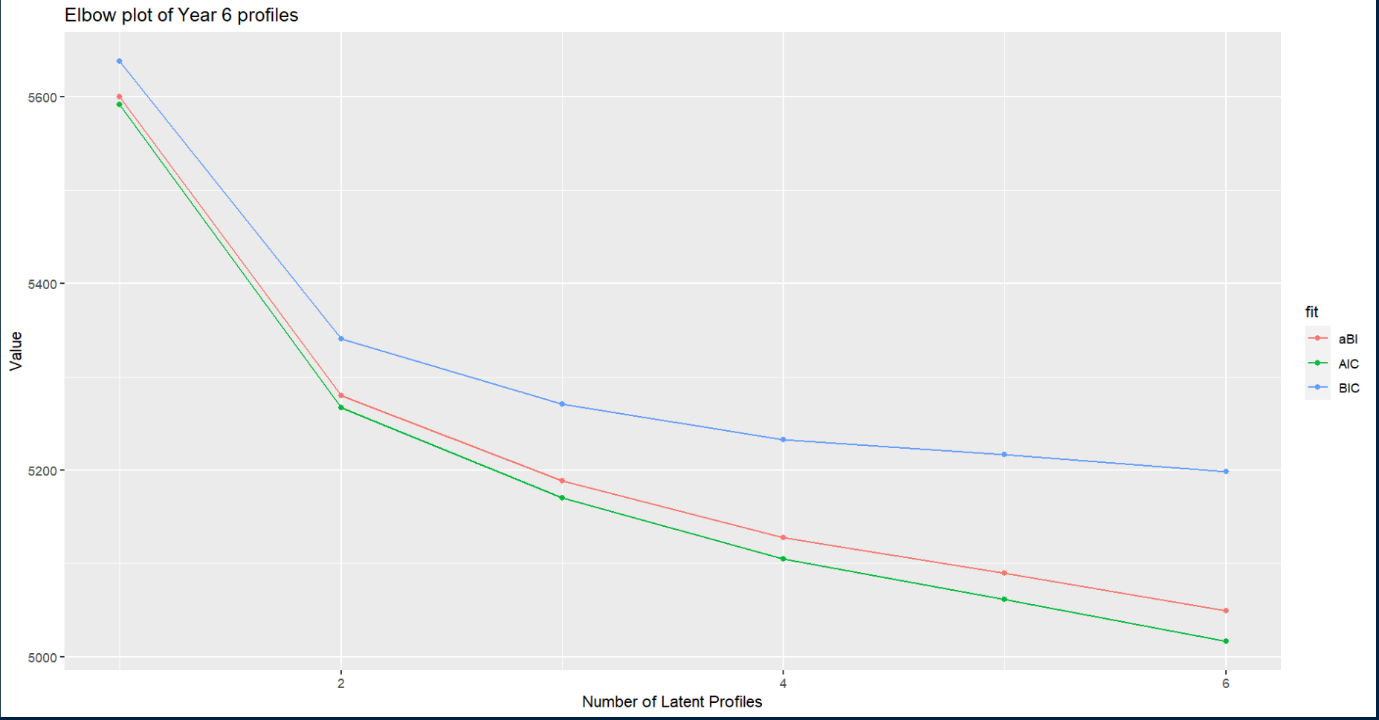

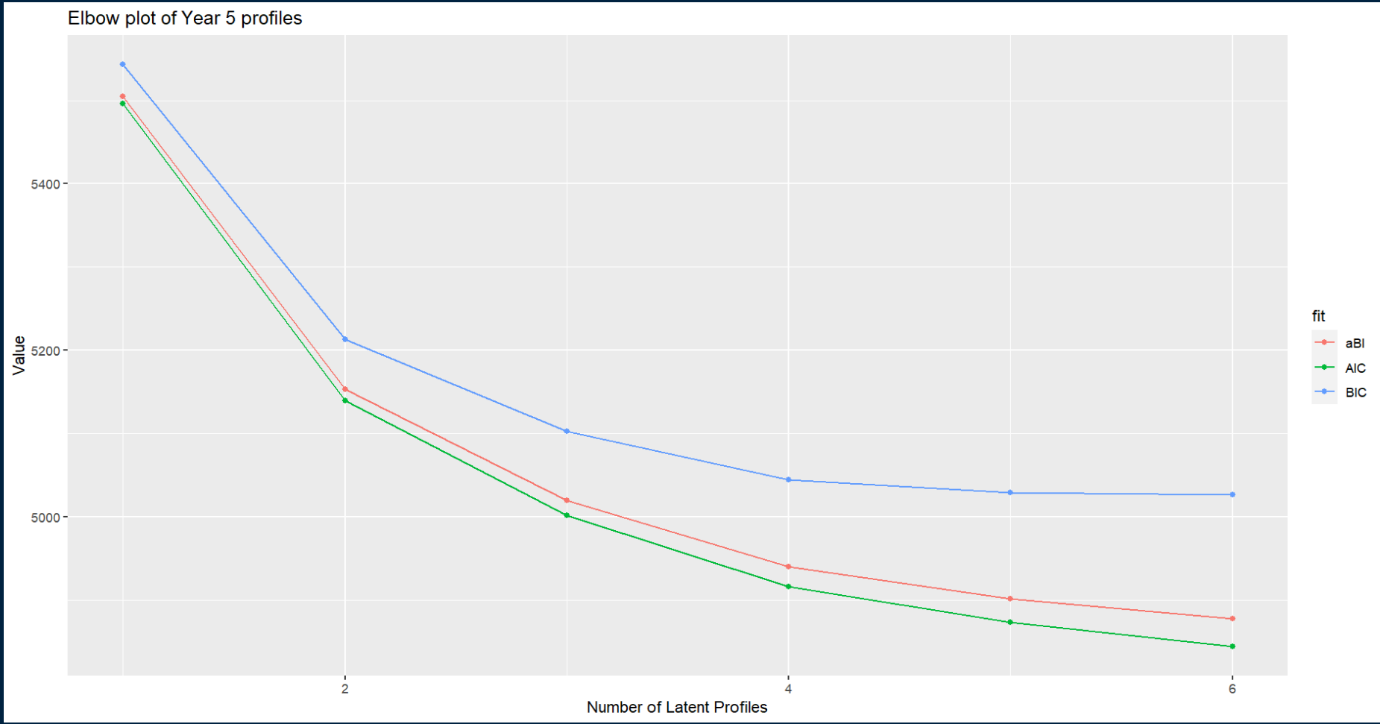


**Supplementary Figure S1.** Elbow plot examining model fit for both grade 5 and 6.

**Supplementary Table S1.** Means and zero-order correlations of each predictor and outcome variable

|  | **Term** | **1** | **2** | **3** | **4** | **5** | **6** | **7** | **8** | **9** | **10** | **11** | **12** | **13** | **14** | **15** | **16** | **17** |
| --- | --- | --- | --- | --- | --- | --- | --- | --- | --- | --- | --- | --- | --- | --- | --- | --- | --- | --- |
|  | **Year 5** |  |  |  |  |  |  |  |  |  |  |  |  |  |  |  |  |  |
|  | ***Science*** |  |  |  |  |  |  |  |  |  |  |  |  |  |  |  |  |  |
| **1** | **SC** | (3.671) |  |  |  |  |  |  |  |  |  |  |  |  |  |  |  |  |
| **2** | **STV** | .560* | (3.579) |  |  |  |  |  |  |  |  |  |  |  |  |  |  |  |
|  | ***Math*** |  |  |  |  |  |  |  |  |  |  |  |  |  |  |  |  |  |
| **3** | **SC** | .325* | .128* | (3.826) |  |  |  |  |  |  |  |  |  |  |  |  |  |  |
| **4** | **STV** | .237* | .430* | .627* | (3.923) |  |  |  |  |  |  |  |  |  |  |  |  |  |
|  | ***Finnish*** |  |  |  |  |  |  |  |  |  |  |  |  |  |  |  |  |  |
| **5** | **SC** | .488* | .219* | .349* | .271* | (3.607) |  |  |  |  |  |  |  |  |  |  |  |  |
| **6** | **STV** | .274* | .489* | .262* | .583* | .578* | (3.670) |  |  |  |  |  |  |  |  |  |  |  |
|  | **Year 6** |  |  |  |  |  |  |  |  |  |  |  |  |  |  |  |  |  |
|  | ***Science*** |  |  |  |  |  |  |  |  |  |  |  |  |  |  |  |  |  |
| **7** | **SC** | .594* | .355* | .236* | .135* | .311* | 0.075 | (3.734) |  |  |  |  |  |  |  |  |  |  |
| **8** | **STV** | .343* | .482* | .072 | .230* | .163* | .220* | .560* | (3.484) |  |  |  |  |  |  |  |  |  |
|  | ***Math*** |  |  |  |  |  |  |  |  |  |  |  |  |  |  |  |  |  |
| **9** | **SC** | .247* | -.057 | .733* | .430* | .294* | .149* | .328* | .210* | (3.755) |  |  |  |  |  |  |  |  |
| **10** | **STV** | .128* | .109 | .387* | .497* | .237* | .342* | .200* | .483* | .622* | (3.747) |  |  |  |  |  |  |  |
|  | ***Finnish*** |  |  |  |  |  |  |  |  |  |  |  |  |  |  |  |  |  |
| **11** | **SC** | .334* | .071 | .243* | .111 | .636* | .336* | .476* | .217* | .336* | .174* | (3.561) |  |  |  |  |  |  |
| **12** | **STV** | .063 | .194* | .060 | .239* | .372* | .508* | .095 | .397* | .188* | .494* | .485* | (3.422) |  |  |  |  |  |
|  | **O*utcome*** |  |  |  |  |  |  |  |  |  |  |  |  |  |  |  |  |  |
| **13** | **Asp5** | -.062 | -.011 | .050 | .154* | .019 | .057 | -.003 | .059 | .070 | .255* | -.003 | .120 |  |  |  |  |  |
| **14** | **Asp6** | .111 | .094 | .072 | .156* | .079 | .098 | .131* | .152* | .118 | .267* | .027 | .043 | .575* |  |  |  |  |
| **15** | **Score_M** | .209* | .005 | .563* | .294* | .209* | .101 | .183* | .125* | .595* | .330* | .182* | .101 | .103 | .152* | (8.337) |  |  |
| **16** | **Score_S** | .309* | .115 | .291* | .195* | .253* | .117 | .349* | .209* | .317* | .189* | .264* | .078 | .075 | .156* | .657* | (8.414) |  |
| **17** | **Score_F** | .257* | .047 | .260* | .093 | .384* | .163* | .312* | .142* | .299* | .117* | .441* | .201* | .118 | .183* | .634* | .718* | (8.354) |
|  | ***Predictor*** |  |  |  |  |  |  |  |  |  |  |  |  |  |  |  |  |  |
| **18** | **sex** | 0.109* | .055 | .278* | .167* | -.059 | -.019 | .081 | -.003 | .145* | .036 | -.107* | -.096 | -.174* | -.139* | .045 | -.163* | -.322* |

*Note: (*) statistically significant, p<.05 | SC = Self-Concept | STV = Subjective Task Value | Asp = STEM Aspiration | M = Math | S = Science | F = Finnish*

**Supplementary Table S2.** Cross tabulation of STEM aspiration (HBMS* vs MPCES*) and its relationship to gender

|  |  |  | ***Gender*** | | |  |  |
| --- | --- | --- | --- | --- | --- | --- | --- |
| ***Aspiration*** |  |  | **Girl** |  | **Boy** |  | **Total** |
| **Year 5** |  |  |  |  |  |  |  |
| Non-STEM | *Count* |  | 97 |  | 74 |  | 171 |
|  | *Std residual* |  | -0.971 |  | 1.256 |  |  |
|  | *Adj std residual* | | -2.777 |  | 2.777 |  |  |
| HBMS* | *Count* |  | 52 |  | 8 |  | 60 |
|  | *Std residual* |  | 2.356 |  | -3.048 |  |  |
|  | *Adj std residual* | | 4.409 |  | -4.409 |  |  |
| MPCES* | *Count* |  | 10 |  | 13 |  | 23 |
|  | *Std residual* |  | -1.159 |  | 1.499 |  |  |
|  | *Adj std residual* | | -1.987 |  | 1.987 |  |  |
|  |  |  |  |  |  |  |  |
| **Total** |  |  | 159 |  | 95 |  | 254 |
|  |  |  |  |  |  |  |  |
| **Year 6** |  |  |  |  |  |  |  |
| Non-STEM | *Count* |  | 81 |  | 71 |  | 152 |
|  | *Std residual* |  | -0.852 |  | 1.013 |  |  |
|  | *Adj std residual* | | -2.194 |  | 2.194 |  |  |
| HBMS* | *Count* |  | 44 |  | 9 |  | 53 |
|  | *Std residual* |  | 2.325 |  | -2.765 |  |  |
|  | *Adj std residual* | | 4.095 |  | -4.095 |  |  |
| MPCES* | *Count* |  | 15 |  | 19 |  | 34 |
|  | *Std residual* |  | -1.102 |  | 1.310 |  |  |
|  | *Adj std residual* | | -1.848 |  | 1.848 |  |  |
|  |  |  |  |  |  |  |  |
| **Total** |  |  | 140 |  | 99 |  | 239 |

**Note: HBMS = Health, Bio and Medical Science | MPCES = Math, Physics, Computer and Engineering Sciences.*

**Supplementary Table S3.** Fit summary of each model

| **Model** | **#fp** | **LL** | **Scaling** | **AIC** | **AICC** | **BIC** | **SABIC** |
| --- | --- | --- | --- | --- | --- | --- | --- |
| *Final cross-sectional Latent Profile Analyses* | | |  |  |  |  |  |
| Y5: 5th grade (4 profiles) | 33 | -2425.365 | 1.372 | 4916.730 | 4923.613 | 5044.971 | 4940.278 |
| Y6: 6th grade (4 profiles) | 33 | -2519.331 | 1.494 | 5104.662 | 5111.631 | 5232.535 | 5127.844 |
|  |  |  |  |  |  |  |  |
| *Longitudinal Latent Profile Analyses* | |  |  |  |  |  |  |
| Configural Similarity | 66 | -4944.696 | 1.433 | 10021.392 | 10044.423 | 10292.75 | 10083.29 |
| Structural Similarity | 42 | -4969.374 | 1.662 | 10022.749 | 10031.602 | 10195.43 | 10062.14 |
| Dispersion Similarity | 36 | -4980.744 | 1.750 | 10033.488 | 10039.923 | 10181.50 | 10067.25 |
| Partial Dispersion Similarity | 42 | -4949.239 | 1.472 | 9982.478 | 9991.331 | 10155.16 | 10021.87 |
| Partial Distributional Similarity | 39 | -4953.224 | 1.515 | 9984.447 | 9992.038 | 10144.79 | 10021.02 |
| *Latent Transition Analysis* | 15 | -676.033 | 0.867 | 1382.066 | 1383.461 | 1440.357 | 1392.769 |
|  |  |  |  |  |  |  |  |
| *Explanatory Analysis* |  |  |  |  |  |  |  |
| Gender | 18 | -668.636 | 0.892 | 1373.272 | 1375.278 | 1443.222 | 1386.117 |
| Achievement: Science | 24 | -1015.437 | 0.877 | 2078.874 | 2082.456 | 2172.140 | 2096.000 |
| Achievement: Math | 24 | -1042.346 | 0.933 | 2132.691 | 2136.273 | 2225.958 | 2149.818 |
| Achievement: Finnish | 24 | -1021.676 | 0.849 | 2091.352 | 2094.934 | 2184.618 | 2108.478 |
| STEM Aspiration | 21 | -853.785 | 0.847 | 1749.570 | 1752.304 | 1831.178 | 1764.555 |
| HBMS-MPCES Aspiration* | 24 | -962.925 | 1.1579 | 1973.851 | 1977.433 | 2067.117 | 1990.977 |

**Note: HBMS = Health, Bio and Medical Science | MPCES = Math, Physics, Computer and Engineering Science.*

**Supplementary Table S4.** Proportion of participants in each latent profile

| **Grade** | **Class** | **Percentage** | **Gender** | **Freq** |
| --- | --- | --- | --- | --- |
| 5 | 1 | 27.22 | Girl | 49 |
|  |  |  | Boy | 49 |
|  | 2 | 51.39 | Girl | 97 |
|  |  |  | Boy | 88 |
|  | 3 | 12.77 | Girl | 36 |
|  |  |  | Boy | 10 |
|  | 4 | 8.61 | Girl | 18 |
|  |  |  | Boy | 13 |
| 6 | 1 | 15.28 | Girl | 27 |
|  |  |  | Boy | 28 |
|  | 2 | 41.67 | Girl | 83 |
|  |  |  | Boy | 67 |
|  | 3 | 9.44 | Girl | 27 |
|  |  |  | Boy | 7 |
|  | 4 | 7.22 | Girl | 14 |
|  |  |  | Boy | 12 |
|  | NA | 26.39 | Girl | 49 |
|  |  |  | Boy | 46 |

**Supplementary Table S5.** Missing values for each variable

| **Variable** | **N** | **Percentage** |
| --- | --- | --- |
| Asp5 | 106 | 29.4 |
| Asp6 | 178 | 49.4 |
| **Science** |  |  |
| SC6 | 95 | 26.4 |
| STV6 | 95 | 26.4 |
| Score | 81 | 22.5 |
| **Math** |  |  |
| SC6 | 95 | 26.4 |
| STV6 | 95 | 26.4 |
| Score | 81 | 22.5 |
| **Finnish** |  |  |
| SC5 | 1 | 0.3 |
| SC6 | 95 | 26.4 |
| STV6 | 95 | 26.4 |
| Score | 83 | 23.1 |
